# Hollow Auxetic Polymer Structures with Manufacturing-Constrained Design and Mechanical Validation

**DOI:** 10.3390/polym18070828

**Published:** 2026-03-28

**Authors:** Finlay Bridge, Rakan Albarakati, Hany Hassanin, Khamis Essa

**Affiliations:** 1 Mechanical Engineering, University of Birmingham, Edgbaston, Birmingham B15 2TT, UK; 2 Industrial Engineering Department, College of Engineering and Architecture, Umm Al-Qura University, Makkah 21955, Saudi Arabia; 3 School of Sciences, Psychology, Arts and Humanities, Computing, Engineering, and Sport, Canterbury Christ Church University, Canterbury CT1 1QU, UK

**Keywords:** auxetic metamaterial, hollow strut, stereolithography, design of experiments, Poisson’s ratio

## Abstract

Hollow auxetic structures enable lightweight mechanical design by reducing mass while preserving architected deformation. However, hollow auxetic studies focus on LPBF metals. This study presents a manufacturing-constrained design and validation framework for a hollow hybrid re-entrant chiral lattice produced by stereolithography. The unit cell was parameterised by chiral angle, re-entrant strut length, and hollow internal diameter, with drainage features integrated into the CAD model to preserve hollow channels during printing and post-processing. A minimum internal diameter study defined the printable design window. Within these limits, a central composite design coupled with finite element analysis mapped the response surface and identified an optimised geometry of θ = 15°, L = 3.5 mm, and d = 1.68 mm, with a predicted unit-cell negative Poisson’s ratio of about −1.17. Compression testing confirmed that the printed unit cell and 3 × 3 × 3 lattice retained the intended rotation-dominated auxetic deformation mode. At the selected comparison strain, the unit cell showed a negative Poisson’s ratio of −0.68 and the 3 × 3 × 3 lattice showed −0.29. Relative to the solid lattice, the hollow lattice reduced density by 42.4% with only a 3.0% reduction in stiffness, increasing specific stiffness by 68.9% and specific peak strength by 5.2%, but reducing specific energy absorption by 25.6% due to earlier localisation and junction driven fracture. These results provide practical design guidance for manufacturable hollow SLA auxetic lattices, especially for lightweight and stiffness-limited applications where low mass and high specific stiffness are more important than energy absorption.

## 1. Introduction

Auxetic lattices are a major class of mechanical metamaterials because their unique behaviour is governed by architecture, not only by the base material [1,2]. This design freedom enables a negative Poisson response through several mechanism families, including re-entrant cells, chiral lattices, and rotating-unit systems, and it allows the deformation pathway to be programmed for a target load case rather than inferred from bulk material behaviour [3,4]. Applications of auxetic lattices include pressure garments for hypertrophic scar treatment, impact absorbers for automobiles, wing fillers for aircraft, and strain sensors, alongside broader use in protective systems and biomedical engineering [5]. A 3D re-entrant auxetic metamaterial study shows how geometric parameters can be used to tune compression stiffness and energy absorption [6]. Member-level geometric engineering can also enhance auxetic response under large deformation by exploiting nonlinear effects in thin members, although this can introduce stiffness trade-offs that must be managed at the design stage [7]. Modified re-entrant auxetic designs fabricated by additive manufacturing have also been assessed for in-plane energy absorption [8]. Recent work on bioinspired, 3D-printable structures shows that geometry-led design can improve compressive performance and energy absorption while keeping mass low [9].

Additive manufacturing has accelerated deployment because many auxetic topologies are difficult to fabricate, using conventional processes such as extrusion and injection moulding [5]. Broader additive manufacturing reviews also highlight the industrial pull for architected structures, driven by design freedom, mass customisation, and rapid prototyping across biomedical, aerospace, construction, and defence applications [10]. Stereolithography (SLA), which is classified as a vat photopolymerisation (VP) process according to ISO/ASTM 52900 [11], is particularly relevant in this context because it offers the geometric resolution needed to manufacture thin-walled hollow struts, drainage features, and controlled auxetic cell geometry. This makes SLA useful for design validation and for low-volume functional polymer components where geometric accuracy is more important than production rate. SLA has also been used to manufacture polymeric re-entrant auxetic lattices for experimental evaluation, with the ability to vary unit cell geometry, scale, and specimen thickness systematically [11,12]. The response of SLA-printed auxetic lattices is sensitive to loading regime and lattice scale, including the number and size of unit cells under quasi-static and dynamic loading [12].

SLA polymer lattices are also sensitive to resin state and post-processing conditions. Guttridge et al. showed in complex vat-photopolymer geometries that extending post-curing duration changed mechanical properties for some biocompatible resins, and that specimen position within the same multi-layer part also affected the measured response, which indicates that generic manufacturer curing guidance can leave non-uniform properties in complex builds [13]. This is directly relevant to lattices because cure non-uniformity across junction-rich geometries can alter the intended deformation path and promote early local failure. Bayarsaikhan et al. also showed in 3D-printed dental resin systems that post-curing temperature affects both mechanical behaviour and biocompatibility, with higher post-curing temperatures improving flexural strength and cell viability in their tests [14]. SLA auxetic response is likewise sensitive to local cell geometry and contact. In the SLA study by Varas et al. on a re-entrant unit cell based on cylindrical elements, both geometric scale and the number of unit cell layers changed the compressive response under quasi-static and SHPB dynamic loading, and the authors linked stiffness and instability to cylinder dimensions and contact between neighbouring elements [12]. Beyond geometry, the effective behaviour of SLA auxetics depends strongly on build-related anisotropy and on the final cure state of the resin. Ermurat and Dag showed that build orientation and loading direction change compressive strength, energy absorption, and auxetic response in re-entrant honeycomb structures produced by AM. Their study also found that DLP-printed specimens behaved more isotropically than FDM specimens, while FDM showed stronger orientation dependence because of interlayer effects. These results reinforce that auxetic performance is process dependent as well as topology dependent [15].

Hollow and tubular members have become an important design route for metal lattice materials because they can provide high structural efficiency at low mass while enabling controlled collapse modes. Laser powder bed fusion, LPBF, has made these architectures practical at relevant lattice densities, and recent work shows that strut cross-section design can be as important as unit cell topology. In Ti-6Al-4V, Noronha et al. fabricated hollow-strut FCC and FCCZ lattices by LPBF and reported yield strength and elastic modulus at the upper empirical limits for solid-strut lattices of similar relative density, while also establishing a manufacturable LPBF route for this class of structures [16]. Earlier hollow-truss studies by Queheillalt and Wadley also showed that hollow members allow independent control of cell size and relative density, with clear relevance to multifunctional aims including load support, thermal management, and dynamic protection [17]. The hollow pyramidal lattice work further showed that out-of-plane compressive strength can be about twice that of comparable solid-truss lattices at similar relative density, linked to increased radius of gyration and improved buckling resistance in hollow members [18].

Hollowing is attractive for impact and crash applications because tubular members can act as distributed thin-walled absorbers and help stabilise post-yield crushing. Xiao et al. experimentally and numerically characterised SLM 316L hollow-strut lattices under quasi-static and dynamic compression and reported stable crushing with smooth post-yield plateaus, together with strong strain-rate sensitivity of strength and energy absorption [19]. Hollow and curving strategies have also been used to improve energy absorption and failure behaviour in additively manufactured lattices. In an SLA octet-truss study, combining hollow cores with varying strut cross-sections increased absorbed energy and stabilised the stress–strain response by making stress distributions more uniform and reducing damage-prone tensile concentrations at nodes [20]. Quasi-static hollow microlattice work also supports the central role of hollow-member buckling and collapse control and shows that gradient thickness or radius can guide buckle propagation and raise energy absorption density by more than 40% [21]. More recent work has moved beyond constant-section hollow struts to Bézier-based nonuniform hollow sections, where the strut profile is deliberately varied to redistribute stress, reduce local stress concentration, and improve stiffness, strength, and toughness relative to benchmark constant-section designs [22].

Even with these advances, metal hollow-strut lattices still face practical constraints that strongly influence what can be realised in design. In powder bed fusion routes, hollow channels must remain connected and sufficiently open for powder evacuation, otherwise partially occluded passages and trapped or adhered powder can undermine dimensional fidelity and repeatability. Internal surfaces can also retain partially sintered particles, which increases roughness and local geometric imperfection in thin members. These manufacturability limits have pushed the field toward open, interconnected hollow networks and toward node designs that reduce stress concentrations while preserving drainage paths and printable feature sizes [16,23]. As a result, much of the state of the art in hollow lattices is concentrated on topologies that combine mechanical efficiency with robust manufacture, rather than on architectures that also require tightly controlled lateral deformation pathways. Within auxetic lattices, hollowing concepts have already been used in metallic re-entrant systems to improve energy absorption, with hollow re-entrant auxetic lattices showing higher specific energy absorption than their solid counterparts at matched relative density [24]. Hollow auxetic cylinder lattice structures have also been characterised under axial compression, where they show a negative Poisson response, energy absorption, and a distinctive rotational deformation mode that can be tuned by geometric parameters and layer count [25]. In addition, re-entrant honeycombs with hollow-circle joints have been proposed to tailor in-plane elastic constants and auxetic response through joint geometry and wall thickness [26].

Recent reviews also show that auxetic lattice performance is most commonly tuned through re-entrant, chiral, and rotating-rigid-unit families, with design methods increasingly combining analytical modelling, numerical optimisation, and manufacturing-aware development [27]. A major step forward has been the use of topology and shape optimisation to programme Poisson’s ratio over meaningful strain windows. Clausen et al. demonstrated topology-optimised architectures with a programmable Poisson’s ratio, then validated printed architectures spanning values from strongly negative to positive, with nearly constant response maintained to large deformation levels of about 20% [28]. Complementary work provides a systematic route to 3D auxetic lattices with a programmable constant Poisson’s ratio at finite strain, including explicit designs covering target Poisson ratio values in the range 0 to −0.78 [29]. These advances confirm that auxeticity can be designed with high precision, yet they still largely assume solid-member architectures. A parallel route is to exploit elastic instability and buckling as the auxetic mechanism in soft metamaterials, which broadens the design space beyond hinge-dominated kinematics [30].

Material distribution provides a further lever that begins to resemble the logic of hollowing without requiring hollow members in the polymer literature. Chen and Zheng demonstrated multi-material additively manufactured metamaterials in which encoded spatial elasticity, from rigid to soft, enables a tailorable Poisson’s ratio from strongly negative to near zero and supports functionally graded strain amplification within a uniform micro-architecture [31]. In parallel, polymer lattice studies show that compressive response and energy absorption in SLA-made structures remain strongly architecture dependent. Wang et al. reported that stereolithography-fabricated polymer lattices exhibit clear differences between uniform and graded designs and between BCC and z-reinforced BCCz variants. In their results, BCCz lattices delivered higher modulus and strength than BCC lattices, while uniform lattices absorbed more energy at small compression distances and graded lattices absorbed more energy at larger compression distances [32]. Bolan et al. showed that SLA octet-truss lattices can transition between brittle and more progressive failure depending on resin type, with relative density and strut geometry shaping both plateau behaviour and absorbed energy [33]. Recent auxetic-specific developments also address practical performance limits such as anisotropy and energy absorption without changing the strut cross-section. Rogers et al. optimised 3D near-isotropic auxetic star cells and demonstrated that symmetry and link design can reduce anisotropy while maintaining a negative Poisson’s ratio [34]. Bastola et al. used strut reinforcement concepts to raise strength and specific energy absorption in 3D re-entrant auxetic lattices under compression [35].

Despite the growing literature on auxetic lattices, a clear gap remains in the development of hollow polymer auxetic lattices produced by SLA. Existing SLA polymer auxetic studies mainly focus on solid lattices, while hollow-strut strategies have been explored more extensively in metal lattice systems. As a result, there is still limited validated evidence on whether a genuinely hollow SLA polymer auxetic lattice can be designed, manufactured, and mechanically validated while preserving the intended auxetic deformation mode. The objective of this study is therefore to investigate whether introducing a controlled hollow core into an SLA-printed hybrid re-entrant chiral auxetic lattice can retain negative Poisson behaviour under compression while improving mass-specific mechanical performance within a manufacturable design space. The main contributions of this work are threefold. First, a printability study is used to define a manufacturable design window for hollow SLA features. Second, a central composite design coupled with finite element analysis is used to map the response surface and identify an optimised hollow auxetic geometry. Third, the optimised design is fabricated and experimentally validated at both unit-cell and 3 × 3 × 3 lattice scales, followed by a direct comparison between hollow and solid lattices in terms of stiffness, strength, and specific energy absorption. These contributions provide a clearer manufacturing-constrained design and validation route for hollow SLA polymer auxetic lattices.

## 2. Materials and Methods

**Figure 1:**
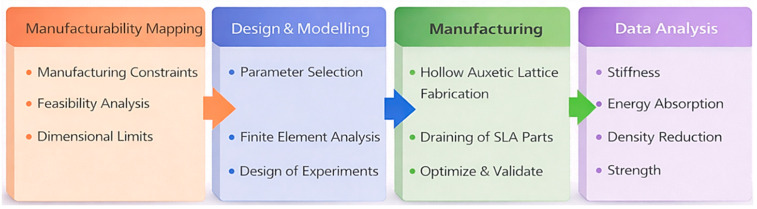
Workflow overview for design, optimisation, manufacture, and validation of the hollow hybrid auxetic lattice.

The study followed the staged design–model–manufacture–test workflow summarised in Figure 1. First, a parameterised hollow hybrid unit cell was defined using three geometric variables. Hollow struts and drainage features were incorporated directly into the CAD to ensure resin evacuation and consistent post-processing during stereolithography. Printability constraints were then established using a minimum internal diameter study, which defined the lower bound for the hollow diameter and therefore restricted the feasible design space used in later optimisation. Within these manufacturable bounds, a central composite design was constructed and evaluated using finite element analysis. Poisson’s ratio was calculated at a common axial strain comparison point, which enabled a consistent comparison across DOE screening and response surface fitting. The resulting response surface was then used to quantify factor significance and interactions and to select an optimised geometry. Finally, the optimised design and selected DOE validation points were fabricated by SLA and tested in uniaxial compression. Image-based strain tracking was used to quantify Poisson’s ratio during loading, and the experimental results were used to compare hollow and solid lattices using both absolute properties and density-normalised metrics.

### 2.1. Unit Cell and Lattice Design

**Figure 2:**
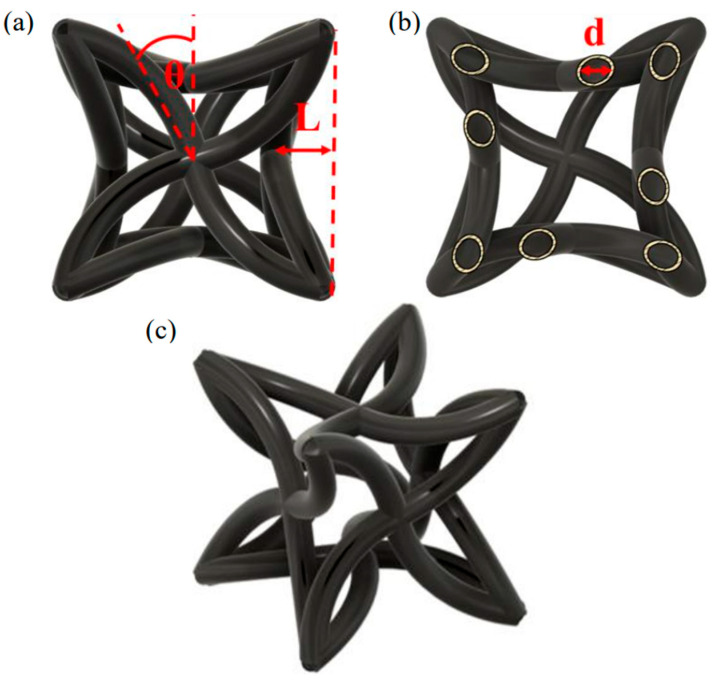
Unit cell design. (**a**) Front view showing the chiral angle θ and re-entrant length L. (**b**) Cross-sectional view showing the internal diameter d of the hollow strut. (**c**) Isometric view of the parameterised unit cell geometry.

The auxetic lattice is constructed from a parameterised hollow hybrid unit cell that combines re-entrant and rotational deformation mechanisms, as illustrated in Figure 2. The unit cell geometry is defined by three primary design parameters: the chiral angle θ, the strut length L, and the hollow strut diameter d. These parameters control the kinematics of cell rotation and hinging under axial compression and therefore govern the magnitude of transverse contraction or expansion observed during loading.

The parameter θ sets the initial inclination of the re-entrant ligaments and determines the degree of rotational freedom available during compression. Smaller values of θ promote greater inward rotation of the struts and are expected to enhance the auxetic response, whereas larger values progressively suppress rotational motion and reduce the effective negative Poisson’s ratio. In the present study, the angle range was limited from 0° to 30° because this range preserves the intended hybrid re-entrant rotational mechanism within a compact and manufacturable geometry. The lower bound of 0° corresponds to the most re-entrant configuration considered in the adopted unit cell definition, while the upper bound of 30° was selected to avoid moving too far away from the targeted auxetic deformation mode. Larger angles such as 45° and 60° were not included in the present work and are identified as a possible direction for future study. The strut length L controls both the aspect ratio of the deforming members and the relative compliance of the unit cell, influencing the balance between bending-dominated and stretching-dominated deformation. The hollow diameter d defines the wall thickness of the struts and therefore directly affects local bending stiffness, stress concentration at the nodes, and overall mass of the lattice.

Hollow struts were adopted to reduce apparent density and to enable systematic evaluation of specific mechanical properties. To ensure successful fabrication by stereolithography, internal drainage features were integrated into the unit cell design, allowing uncured resin to be evacuated from the hollow cores during post-processing. These drainage features were incorporated at the CAD stage and treated as an intrinsic part of the unit cell topology rather than as post hoc modifications. This approach ensured that all designs considered in the subsequent modelling and optimisation stages were directly manufacturable within the constraints of the printing process.

Periodic arrays of the unit cell were assembled to form three-dimensional lattices for numerical and experimental evaluation, as shown in Figure 2b. The lattice configuration preserves geometric continuity at the cell interfaces, enabling the investigation of both isolated unit cell behaviour and collective lattice-scale deformation and failure mechanisms within a consistent geometric framework.

### 2.2. Printability Constraints and Manufacturability Window

Hollow struts were introduced to reduce apparent density and enable assessment of density-normalised performance. In stereolithography, however, hollow channels are limited by the minimum resolvable feature size and, critically, by the ability to clear uncured resin from enclosed voids during washing and post-processing. To ensure that all candidate designs explored in the optimisation stage were manufacturable, an experimental printability study was conducted to establish a process-specific manufacturability window for hollow features.

**Figure 3:**
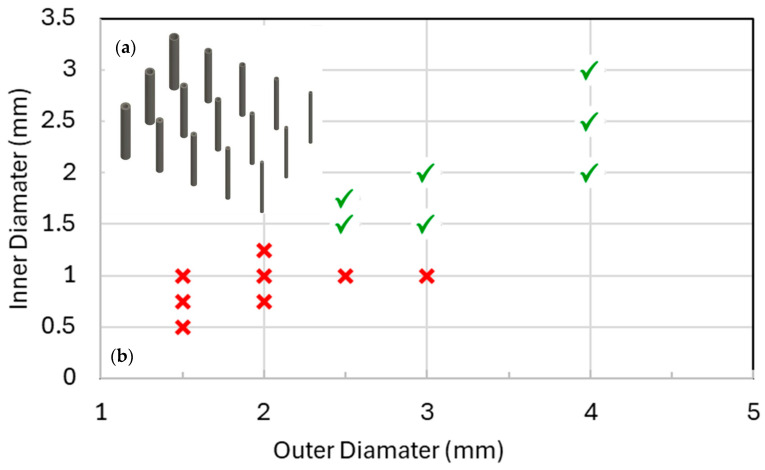
(**a**) Representative hollow tube models used for the printability study. (**b**) Manufacturability envelope in the D–d space showing successful and failed prints and the derived minimum printable internal diameter boundary. (✔ through struts, **x** blocked struts).

The printability study used hollow tube specimens with systematically varied outer diameter, D, and internal diameter, d, fabricated using the same printer, resin, build orientation, and cleaning and curing workflow adopted for the lattice specimens. Representative printed tubes are shown in Figure 3a. After printing, parts were washed and post-processed as per the lattice protocol, and each tube was assessed for successful formation of a continuous hollow channel and for clearance of trapped resin. A print was recorded as successful when the internal channel remained open along the specimen length and could be cleared without visible obstruction or closure after post-curing. Failed samples typically exhibited blocked channels, incomplete formation of the internal void, or resin entrapment that prevented full evacuation.

The outcomes are reported in Figure 3b, which presents the experimentally derived manufacturability envelope. Successful and failed prints are plotted as discrete test points in the D–d design space, and the boundary indicates the minimum printable internal diameter for each tested outer diameter under the selected process conditions. The envelope shows that printability is not governed by a single universal minimum feature size but varies with the external tube diameter. In practical terms, the feasible region lies above the boundary in Figure 3b and defines the internal diameters that can be produced reliably and cleared effectively for a given outer diameter. This manufacturability window was then used to constrain the hollow geometry in the unit cell study. Specifically, the hollow strut diameter parameter d was restricted to values within the feasible region identified by the tube tests. By embedding this experimentally determined constraint into the design space prior to the DOE–FEA stage, all parameter combinations evaluated numerically remained directly manufacturable, reducing the likelihood of optimisation converging to non-printable designs and ensuring that numerical trends were transferable to the fabricated lattices.

### 2.3. Design of Experiments

A structured design of experiments was used to quantify how the unit cell geometry influences the auxetic response and to enable optimisation within a manufacturable design space. Three geometric parameters were selected as DOE factors, namely the chiral angle θ, the re-entrant strut length L, and the internal diameter of the hollow strut d, as defined in the unit cell geometry shown in Figure 2. The factor bounds were selected to preserve the intended deformation mechanism of the hybrid unit cell while remaining compatible with stereolithography fabrication. For the chiral angle, the selected range of 0° to 30° was chosen to focus the DOE on the low-angle regime, where the present topology is expected to retain stronger re-entrant rotation and a clearer auxetic response. Angles above this range were not included because the aim of the study was to optimise the design within a mechanism-relevant and manufacturable window rather than to perform a full parametric sweep of all possible angle values. In particular, the hollow diameter parameter d was constrained using the manufacturability window, ensuring that all designs evaluated were printable and could be cleared of uncured resin.

The selected factors, bounds, and centre values are summarised in Table 1. A central composite design was adopted to efficiently sample this constrained space while enabling estimation of linear effects, second-order curvature, and interaction terms between parameters. The design comprised factorial points to resolve primary trends, axial points to capture curvature in the response, and repeated centre points to provide an estimate of numerical variability and support robust response surface fitting. In the present study, the 20 DOE points were evaluated numerically using finite element analysis of all configurations. For this reason, the DOE dataset in Table 2 represents deterministic numerical outputs.

The DOE response variable was Poisson’s ratio, calculated from the ratio of transverse strain to axial strain under uniaxial compression. Poisson’s ratio was extracted at yield strain to provide a consistent reference point within the linear deformation regime and to minimise the influence of non-linear deformation and contact effects at higher strains. For each DOE design point, the corresponding geometry was generated from the parametric CAD model and evaluated using finite element analysis under identical loading and boundary conditions. Axial and transverse strains were obtained from the simulated displacement fields using consistent virtual gauge locations, and the resulting Poisson’s ratio value was recorded for subsequent response surface modelling and optimisation.

Following evaluation of the DOE set, a response surface model was constructed to relate Poisson’s ratio to θ, L, and d across the constrained design space in Table 1. This model was used to quantify the relative importance of each factor and to identify interaction effects, providing a systematic basis for selecting an optimised parameter set. In addition to the predicted optimum, a subset of DOE cases spanning the design space was selected for fabrication and experimental validation, enabling the direct comparison of numerical predictions with measured auxetic response in printed specimens.

The statistical significance of the response surface model was evaluated using analysis of variance. A quadratic model was fitted to the Poisson’s ratio response, and the effects of the linear, square, and two-way interaction terms were assessed using *p*-values. Terms with *p* < 0.05 were considered statistically significant. Because the repeated centre-point simulations produced identical responses, the pure error was zero and the lack-of-fit test could not be estimated in the usual way.

### 2.4. Finite Element Modelling

**Figure 4:**
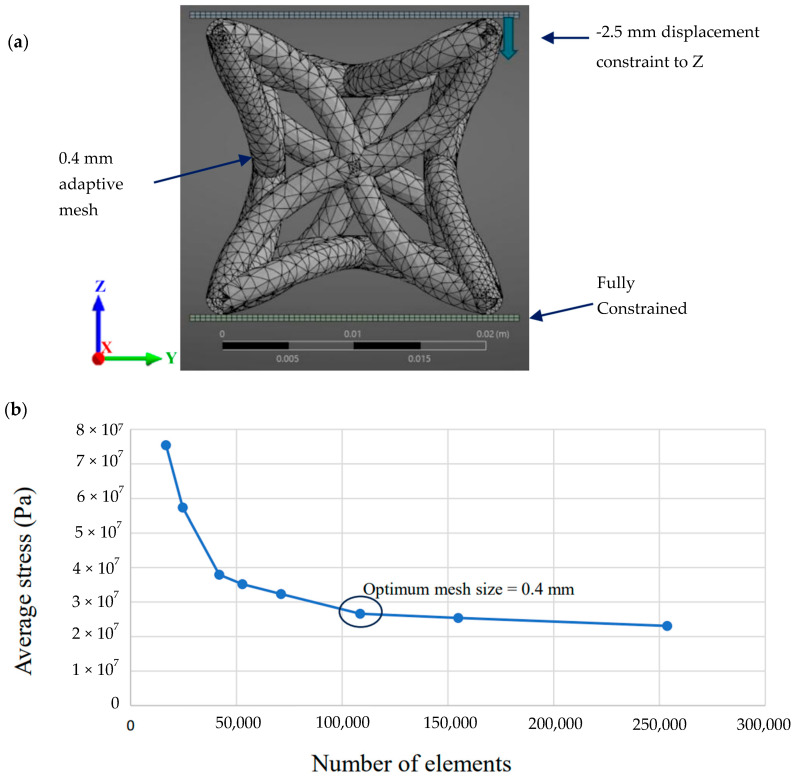
(**a**) FEA set-up and boundary conditions. (**b**) Mesh convergence analysis.

Finite element analysis was used to evaluate the auxetic response of each DOE geometry under uniaxial compression using a consistent numerical workflow. All simulations were performed in Ansys Mechanical 2024 R2 using a Static Structural analysis. For each DOE design point, the corresponding unit cell geometry was generated from the parametric CAD definition and imported into Ansys so that only θ, L and d varied between runs. The full boundary condition set-up is shown in Figure 4a. The unit cell was placed between two rigid plates. The bottom plate was assigned a fixed support, and the top plate was prescribed a 2.5 mm displacement in the z-direction. The top plate was also constrained in x and y, ensuring the load was applied axially while permitting the unit cell to deform laterally within the platen constraint.

The plates were modelled as steel, and bonded contact was applied between the unit cell and the plates. Although frictional contact would be more representative of the experimental interface, bonded contact was adopted because frictional contact produced impractical solve times and instability when repeated across the full DOE set. Meshing was carried out using an adaptive sizing mesh with quadratic elements, and a convergence analysis was undertaken to determine an appropriate global element size. The mesh refinement was continued until the average stress converged, yielding an optimum global element size of 0.4 mm, as summarised in Figure 4b. This mesh specification was then held constant for all DOE simulations to ensure that differences in predicted response were driven by geometry rather than discretisation.

The unit cell material was defined as Formlabs Standard V4 resin, modelled as a homogeneous isotropic solid. The Young’s modulus and Poisson’s ratio used in the model were taken from the resin compression characterisation described elsewhere in the study. The simulations were executed using a linear elastic material model as a consistent comparative tool for DOE screening and optimisation, which reduced computational time while preserving relative ranking across the design space. Poisson’s ratio was calculated from the longitudinal and lateral strain outputs using the study’s definition. The resulting values were used as inputs to the response surface modelling and optimisation stage. The finite element model was intended as a consistent comparative tool for DOE screening and optimisation, rather than as a full failure-predictive model. The bonded platen contact, linear material response at the extraction strain, and idealised geometry are expected to preserve ranking across designs but to overestimate the absolute auxetic magnitude relative to experiment.

### 2.5. SLA Fabrication and Post-Processing

All physical specimens were fabricated using stereolithography to realise the fine hollow features and thin walls required by the unit cell design. SLA offered the resolution needed to manufacture the scaled unit cell at a length of 22.5 mm with a minimum wall thickness of 0.25 mm, using a layer thickness of 0.1 mm. Support strategy was treated as a primary manufacturing requirement because any residual internal supports would alter the deformation mechanism and bias the measured auxetic response. The build orientation and support layout were therefore selected to ensure that no supports were generated inside the hollow members. Only external supports were permitted, and the geometry was orientated to maintain clear internal channels during printing.

Formlabs Standard V4 resin was used for all prints, and specimens were tested in a partially cured state. This reduced stiffness relative to fully cured resin and allowed greater deformation before fracture, which improved the ability to characterise auxetic behaviour across designs. The optimised design was fabricated both as a single unit cell and as a 3 × 3 × 3 lattice for subsequent mechanical testing. The 3 × 3 × 3 configuration was selected as the first finite-lattice validation case because it allowed a direct comparison with the isolated unit cell while remaining practical for stereolithography fabrication, preservation of the hollow features, image-based strain tracking, and observation of localised failure. Maintaining truly hollow struts required reliable resin evacuation. Drainage holes were integrated into the unit cell geometry and placed at the corners of the unit cell, as shown in Figure 2c, to promote outflow from internal cavities during post-processing. Additional drainage features were then introduced after initial print trials to further reduce the likelihood of trapped resin and to preserve the hollow strut architecture.

### 2.6. Mechanical Testing

**Figure 5:**
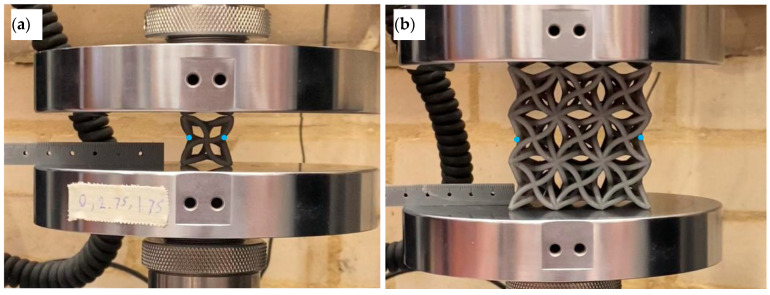
Compression testing. (**a**) Single unit cell between platens with lateral gauge points. (**b**) 3 × 3 × 3 lattice under uniaxial compression with corresponding gauge points for transverse strain extraction.

Uniaxial compression tests were carried out using an Instron 30 kN Universal Testing Machine (Instron, Norwood, MA, USA) to characterise the mechanical response of the printed structures and to quantify Poisson’s ratio during loading. The experimental setup is shown in Figure 5, which includes both the optimised 3 × 3 × 3 lattice and the single unit cell configurations. Each specimen was placed between two flat compression platens. A small preload was applied to ensure contact between the platens and the specimen, then the force and displacement channels were zeroed. To enable accurate measurement of lateral deformation, a video camera on a tripod was positioned so that the optical axis was normal to the specimen.

A known reference distance was placed in the same plane as the lateral deformation to reduce parallax error during image processing. Specimens were first compressed to the selected comparison point at a displacement rate of 0.16 mm s^−1^ for consistent Poisson’s ratio evaluation. The test was then restarted and continued to failure and beyond to capture full stress–strain behaviour and post-failure response. This protocol was applied to five unit cell designs selected from the DOE and to two optimised lattices, one with hollow struts and one with solid struts. Poisson’s ratio was calculated as the ratio of lateral strain to axial strain, using the study definition $\nu =-\varepsilon_{\mathrm{lat}}/\varepsilon_{\mathrm{long}}$.

The DOE validation stage was designed primarily to assess trend agreement and ranking between the numerical model and the printed specimens, rather than to establish a full statistical distribution for each design. For the final hollow versus solid comparison, the reported values are treated as comparative performance indicators for the optimised geometries under identical test conditions. Lateral deformation was obtained from the recorded videos using ImageJ (National Institutes of Health, Bethesda, MD, USA). The reported Poisson’s ratio values were calculated from calibrated ImageJ tracking of predefined gauge points in the recorded test videos, while the images presented later are included only to illustrate the deformation mode. The measurement points used to track lateral displacement were defined consistently for all specimens and are indicated in Figure 5. Axial strain was calculated from the crosshead displacement normalised by the initial specimen height. Lateral strain was calculated from the change in measured lateral distance between the tracked points, normalised by the initial lateral distance. Engineering stress–strain data were extracted from the Instron force and displacement outputs. Stress was calculated using the apparent loaded area of each unit cell or the 3 × 3 × 3 lattice specimen, which provides a consistent basis for comparing designs with different internal geometry. Energy absorption was calculated as the area under the stress–strain curve over the strain interval of interest. For density-normalised comparisons between the hollow and solid lattices, the apparent density was calculated as mass divided by the total volume of the bounding cube, in line with the apparent-density definition used in this study.

## 3. Results

### 3.1. Resin Material Properties

The mechanical response of the partially cured Formlabs Standard V4 resin was characterised in uniaxial compression using a 20 × 20 × 20 mm solid cube. The resulting properties were used to define the resin as a homogeneous isotropic material in the finite element model, so that predicted changes in auxetic response across the design space were driven by geometry rather than assumed material values.

**Figure 6:**
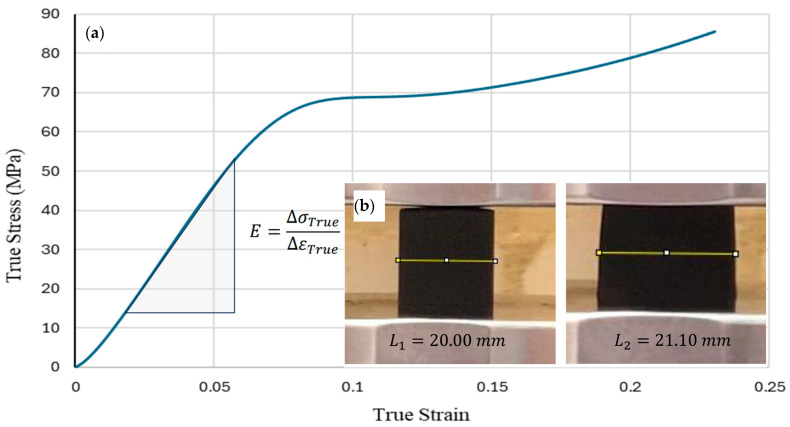
Material property results for Formlabs Standard V4 resin, with (**a**) the stress–strain curve and (**b**) the Poisson’s ratio measurement.

Figure 6a shows the compressive stress–strain behaviour. The response is approximately linear up to about 6% strain. The Young’s modulus calculated from this region is 0.938 GPa. Beyond this, the resin yields at around 9% strain, followed by a plateau and subsequent strain hardening. No fracture was observed within the test end criteria. This ductile response is consistent with testing in a partially cured state, which reduces cross-link density compared with fully cured resin and therefore increases plasticity.

Poisson’s ratio was obtained from lateral deformation of the cube during compression, as illustrated in Figure 6b. The measured value for the partially cured resin was 0.40. Taken together, these values imply a shear modulus of about 0.335 GPa and a bulk modulus of about 1.56 GPa, which are useful checks for numerical stability when defining an isotropic elastic material. These parameters were applied directly in the finite element simulations. From a modelling perspective, using experimentally measured stiffness and Poisson coupling is important because the auxetic response in this study is governed by the bending and rotation of slender members. Small shifts in the base material stiffness can change contact onset and local stress concentrations, which can then bias the predicted Poisson’s ratio. For this reason, the experimentally measured resin properties were used consistently across all DOE simulations.

### 3.2. Simulation and DOE Validation

The DOE was evaluated using finite element analysis for 20 parameter combinations spanning the full design space. The resulting Poisson’s ratio values are summarised in Table 2. Across the DOE set, the predicted Poisson’s ratio ranged from −0.389 to −1.197, with a mean of −0.888, confirming a consistently auxetic response while also showing strong tunability through geometry. Clear geometric trends are evident in Table 2. Increasing the re-entrant length L produced a markedly more negative Poisson’s ratio for most parameter combinations. For example, at θ = 0° and d = 1.5 mm, increasing L from 1.0 mm to 4.5 mm shifts Poisson’s ratio from −0.453 to −1.175. A similar trend is observed at d = 2.0 mm, where Poisson’s ratio changes from −0.389 to −1.052. In contrast, changing the hollow diameter d has a smaller influence within the tested range. At θ = 15° and L = 2.75 mm, increasing d from 1.5 mm to 2.0 mm shifts Poisson’s ratio from −1.035 to −0.920. These comparisons indicate that L dominates the response within the explored space, while d provides secondary tuning.

To validate the numerical and DOE predictions, five unit cells were fabricated and tested in compression, and their measured Poisson’s ratios were compared against the DOE model predictions. The comparison showed good trend agreement, with an R^2^ of 0.7815. However, the regression slope is 0.45, indicating a systematic reduction in auxetic magnitude in the experiments compared with the predicted response. The deviation increases for designs with more negative predicted Poisson’s ratios, meaning the model captures the ranking of designs more reliably than the absolute magnitude at the high-NPR end.

### 3.3. Parameter Significance, Interactions, and Optimisation

Table 3 summarises the key ANOVA results for the quadratic response surface model of Poisson’s ratio. Among the linear terms, only the re-entrant length L was significant (*p* = 0.000), while the chiral angle θ (*p* = 0.156) and inner diameter d (*p* = 0.068) were not significant as individual main effects. However, all three-square terms were significant, showing that the geometric effects were nonlinear. Among the two-way interactions, only θ × L was significant (*p* = 0.000), which indicates that the influence of angle depends strongly on the selected strut length. These findings confirm that L is the dominant design variable, while θ contributes mainly through its interaction with L and through nonlinear curvature in the fitted response.

The response surface model was used to quantify the relative influence of the three geometric variables on Poisson’s ratio and to identify any statistically meaningful interactions. Across the DOE space, the re-entrant length L was the only significant main effect, with *p* = 0.000. In contrast, the hollow internal diameter d and chiral angle θ were not significant within the tested ranges, with *p* = 0.068 and *p* = 0.156, respectively.

**Figure 7:**
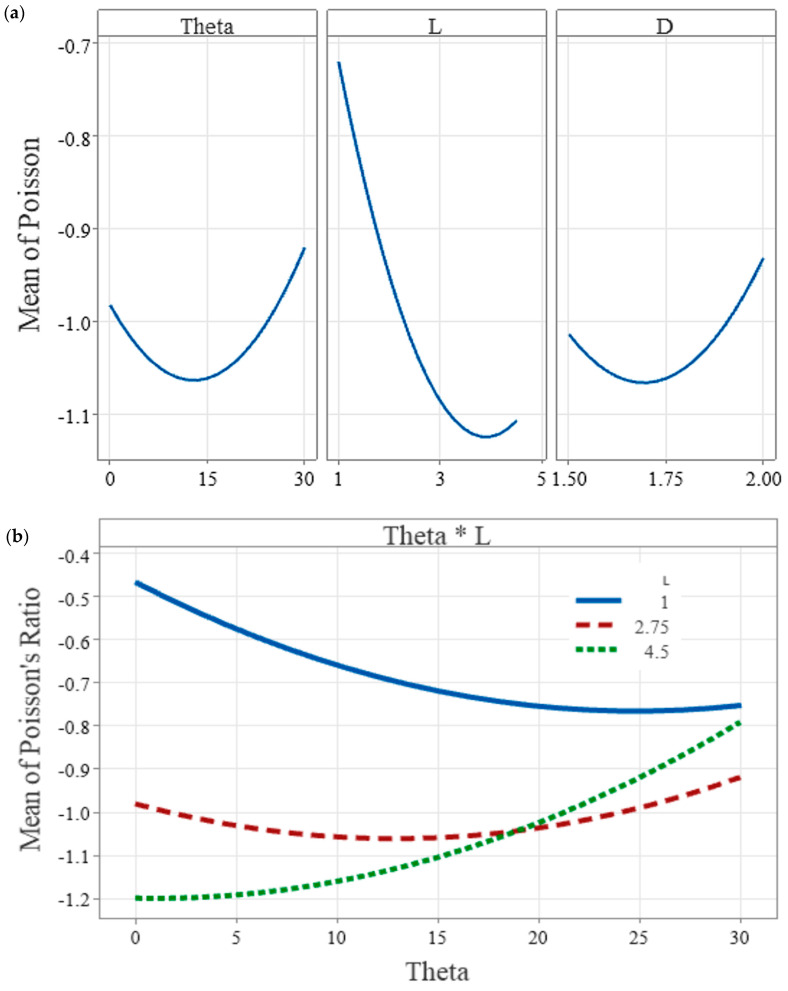
(**a**) Main effects plot for Poisson’s ratio with fitted means. (**b**) θ–L interaction plot with fitted means.

The main-effect trends are shown in Figure 7a. The fitted mean response for L becomes strongly more negative as L increases, before flattening and then rising slightly at the upper end of the range. The fitted means for θ and d show weaker curved trends, but the magnitude of these changes is not large enough to be statistically significant across the explored design space. Only one interaction term was significant, namely the θ–L interaction, with *p* = 0.000. This interaction is shown in Figure 7b. The fitted curves are clearly non-parallel and cross, which indicates that the effect of θ depends on the value of L. For lower L values, the fitted mean Poisson’s ratio is highest at the ends of the θ range and lowest at a mid-range θ. For the highest L level, the trend reverses and Poisson’s ratio increases steadily with θ. This means that at a larger L a smaller θ is preferred, while at a smaller L a mid-range θ becomes more favourable.

Based on these trends, optimisation was carried out to maximise the magnitude of the negative Poisson’s ratio while remaining within the manufacturable domain for SLA printing without internal supports. Using the constrained optimisation range θ = 15 to 30 degrees, L = 1.5 to 3.5 mm, and d = 1.5 to 2.0 mm, the optimised parameter set was θ = 15 degrees, L = 3.5 mm, and d = 1.68 mm with a predicted Poisson’s ratio of −1.17.

### 3.4. Auxetic Deformation and Failure: Unit Cell Versus Lattice

**Figure 8:**
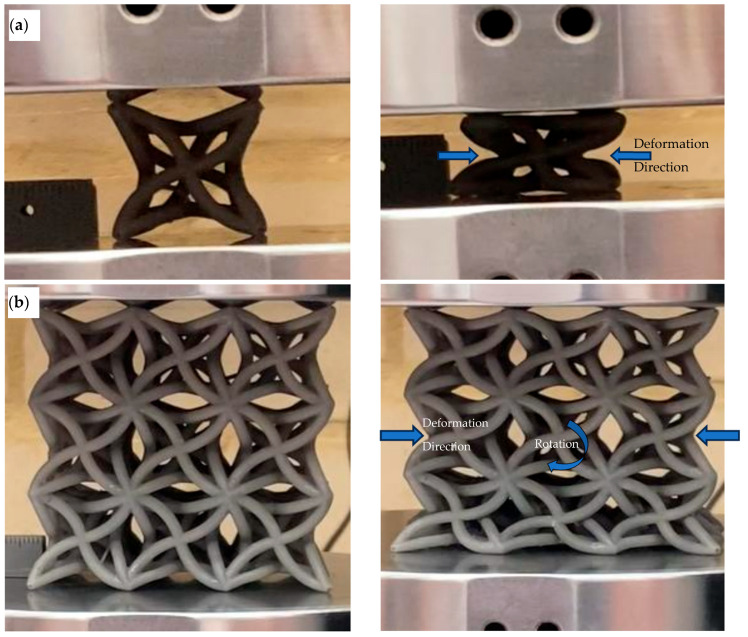
Deformation and failure of the optimised hollow auxetic design. (**a**) Single unit cell. (**b**) 3 × 3 × 3 lattice showing deformation localisation in the bottom row.

The deformation behaviour of the optimised unit cell and the optimised 3 × 3 × 3 lattice is shown in Figure 8a,b. In both cases, the deformation mode is consistent with the finite element predictions. The central region moves inwards during compression and the cell centres rotate slightly clockwise. This confirms that the intended hybrid auxetic mechanism is retained after manufacture.

A clear scale effect is observed when the unit cell is arrayed into a lattice. At the common comparison point of axial strain, the measured negative Poisson’s ratio is −0.68 for the single unit cell and −0.29 for the 3 × 3 × 3 lattice. This corresponds to a 57.4% reduction in auxetic magnitude when moving from the isolated cell to the lattice assembly. The lattice therefore preserves the same deformation pattern, but with a lower auxetic intensity at specimen scale. This result demonstrates that finite-lattice effects are already important in the present structure, although only one lattice size was examined here.

A comparison between the optimised unit cell prediction and the measured responses for the unit cell and 3 × 3 × 3 lattice provides useful context for the observed auxetic behaviour. The DOE-coupled finite element model predicted an NPR of approximately −1.17 for the optimised unit cell. Experimentally, the optimised unit cell exhibited an NPR of −0.68 at the selected comparison point, while the 3 × 3 × 3 lattice exhibited −0.29 at the same comparison point. Together, these results show a clear reduction in realised auxetic response as the structure scale increases, although the intended deformation mechanism is preserved. At the selected comparison point, the 3 × 3 × 3 lattice retains about 42.6% of the unit cell auxetic magnitude, corresponding to a 57.4% reduction. The same trend is seen in the peak response. The single unit cell reaches a maximum negative Poisson’s ratio of −1.1, whereas the 3 × 3 × 3 lattice reaches about −0.4 before fracture. This corresponds to a 64% reduction in peak auxetic magnitude. These results show that the unit cell mechanism remains effective in principle, but the realised lattice response is strongly limited by load redistribution and early structural failure. The localisation of deformation in the lattice is visible in Figure 8a,b, where the bottom row of cells carries the highest deformation. This localised response is consistent with the early loss of lattice-scale auxetic performance and the onset of structural failure. Detailed post-failure fracture features are presented in the Discussion, where they are used to interpret the localisation and failure mechanism.

### 3.5. Hollow Versus Solid Lattice

**Figure 9:**
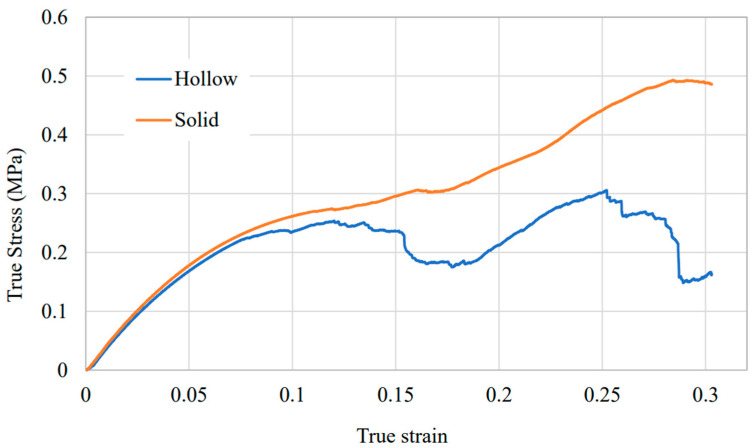
Compressive stress–strain curves for the optimised hollow and solid 3 × 3 × 3 lattices tested under identical conditions.

The hollow and solid optimised 3 × 3 × 3 lattices were tested under the same compression conditions to isolate the effect of strut hollowness on mechanical response. The absolute stress–strain curves are shown in Figure 9. Both structures show a very similar initial elastic region, with Young’s modulus values of 3.22 MPa for the hollow lattice and 3.32 MPa for the solid lattice. This is only a 3.0% reduction in absolute stiffness for the hollow lattice despite the much lower mass. After the elastic region, the responses diverge. Both curves leave the linear regime at around 5% strain and then enter a plateau region. The hollow lattice shows lower stress and more oscillation in this plateau, which is consistent with progressive local fracture and instability in thin-walled members. The first major failure occurs at 15.4% strain, marked by a sharp stress drop. Two further failure events occur at about 25% and 28% strain, which are consistent with subsequent fracture of the upper rows as deformation propagates through the lattice.

By contrast, the solid lattice does not show catastrophic failure within the tested strain range. Instead, the stress rises progressively during densification as internal void spaces close and strut-to-strut contact increases. This difference in post-yield behaviour shows that hollowing preserves stiffness efficiently at low strain but shifts the failure mode towards earlier brittle fracture at node and crossover regions. The peak compressive strength of the solid lattice is 0.507 MPa, compared with 0.306 MPa for the hollow lattice. This is a 39.6% reduction in absolute peak strength for the hollow design. However, the hollow lattice also has a much lower apparent density, 125 kg m^−3^ versus 217 kg m^−3^ for the solid lattice, which is a 42.4% reduction in density. Once density is accounted for, the performance ranking changes.

Density-normalised metrics show the benefit of the hollow design for lightweight applications. The hollow lattice has a specific Young’s modulus of 25,777 m^2^ s^−2^, compared with 15,258 m^2^ s^−2^ for the solid lattice. This is a 68.9% improvement in stiffness per unit mass. The specific peak compressive strength is also higher for the hollow lattice, 2450 m^2^ s^−2^ versus 2330 m^2^ s^−2^, which is a 5.2% improvement. These results support the central design rationale of the study. Hollowing reduces absolute load capacity but improves stiffness efficiency and slightly improves strength efficiency when normalised by density. The specific energy absorption follows a different trend. Table 4 shows a summary of the key results. The hollow lattice gives 303 J kg^−1^, while the solid lattice gives 407 J kg^−1^, so the hollow design is lower by 25.6%. This reflects the earlier fracture of the hollow lattice and the lower capacity for stable progressive crushing before densification. In other words, the present hollow design is most promising for lightweight and stiffness-limited applications, where mass-efficient stiffness and strength are more important than energy absorption. For energy-absorbing applications, further redesign of the node and crossover regions would be needed to delay fracture and improve progressive collapse.

## 4. Discussion

The results show that the optimised geometry preserves the intended auxetic kinematics after fabrication, while the measured response changes in a physically meaningful way when the structure is scaled from a single cell to a finite lattice. The inward motion and rotation-dominated deformation seen in Figure 8a,b match the established behaviour of auxetic mechanical metamaterials [1] and are consistent with the broader interpretation of Poisson’s ratio in engineered materials, where the measured value depends on structural scale and deformation mode as well as material properties [35]. The observed mechanism also follows the classical re-entrant response reported by Lakes, in which bending and rotation of inclined members drive lateral contraction under compression [36]. In the present lattice, the mechanism is not lost after printing. Instead, its expression is redistributed by finite-lattice mechanics.

The DOE and finite element results in Table 2 are important because they define the geometric response surface before fabrication and show that the selected design sits in a strongly auxetic region of the constrained design space. The optimised geometry produced a predicted unit cell NPR of about −1.17, while the experiments gave −0.68 for the unit cell and −0.29 for the 3 × 3 × 3 lattice at the selected comparison point. The unit cell result confirms that the hollow geometry itself has a strong auxetic capability. The lattice result confirms that the same mechanism remains active after scale-up and manufacture. The progression from the predicted unit cell response to the measured unit cell and lattice responses shows that the realised response is controlled by structural effects that are not fully captured in an idealised CAD model. This pattern is consistent with polymer additive manufacturing studies in which FEA reproduces trends and ranking, but the magnitude of the response becomes increasingly sensitive to joint compliance, local geometry, and fabrication-induced variability as the architecture becomes thinner and more bending-dominated [37]. In that sense, the DOE and FEA workflow remains highly effective for screening and optimisation, but the present results show that lattice-scale validation is essential when the design objective depends on a kinematic mechanism.

**Figure 10:**
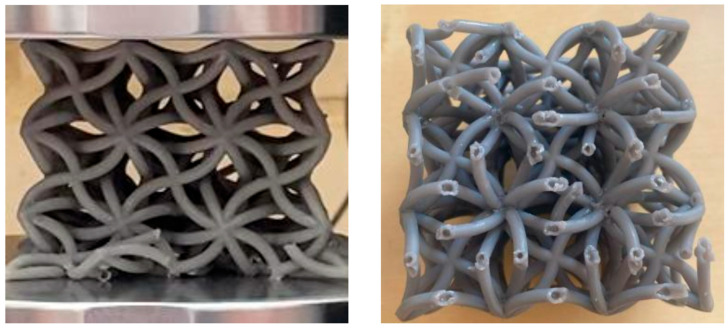
Fracture localisation and hollow-strut integrity in the hollow auxetic lattice after compression.

The reduction in auxetic magnitude from unit cell to lattice is not only a numerical mismatch. It is directly supported by the deformation sequence in Figure 8b and the fracture evidence presented in Figure 10. At the selected comparison point, the lattice retains about 42.6 percent of the unit cell auxetic magnitude, which means that more than half of the local auxetic response is not expressed in the global lattice measurement. The images show deformation localises in the bottom row of cells before the rest of the lattice can develop the same level of coordinated rotation. Once this happens, the transverse strain measured across the specimen becomes a global average of cells with very different kinematics. The measured lattice NPR therefore decreases even though the active cells still deform auxetically. This interpretation is well aligned with size and edge effects reported for lattice-structured cellular materials, where finite boundaries and truncated edge cells alter the effective response measured at specimen scale [38]. It also agrees with studies showing that boundary constraints can suppress the magnitude of negative Poisson’s ratio in auxetic metamaterials by restricting the rotational degrees of freedom needed for full mechanism development [39]. More recent finite-size metamaterial modelling reaches a similar conclusion by showing that explicit boundary and interface terms are needed to recover the response of finite specimens [40]. The size-dependent Poisson’s ratio observed in polymeric lattices with Cosserat-type effects also supports the same interpretation because it shows that characteristic lengths and topology can influence the measured auxetic response when the specimen dimensions are limited [41]. At the same time, the present study only examined one lattice size, namely 3 × 3 × 3. The current results therefore establish the existence of a clear scale effect between the isolated unit cell and a finite lattice, but they do not provide a complete size-dependence map. Future work should extend the study to larger arrays such as 5 × 5 × 5 and 7 × 7 × 7 to quantify how boundary effects, load redistribution, and deformation localisation evolve with increasing lattice size.

The fracture path in Figure 10 provides a second layer of explanation and is central to understanding why the lattice does not approach the optimised numerical value. This fracture pattern also helps explain the lower specific energy absorption of the hollow lattice. Energy absorption depends not only on peak load, but also on the ability of the structure to sustain progressive crushing over a wider strain range. In the present hollow architecture, cracking begins at mechanically critical node and crossover regions before a stable crushing front can fully develop. Once these local failures occur, the structure sheds load through discrete fracture events rather than maintaining a smooth plateau response. This interrupts the continuous dissipation of strain energy and contributes directly to the lower SEA measured for the hollow lattice. This fracture pattern also helps explain the lower specific energy absorption of the hollow lattice. Energy absorption depends not only on peak load, but also on the ability of the structure to sustain progressive crushing over a wider strain range. In the present hollow architecture, cracking begins at mechanically critical node and crossover regions before a stable crushing front can fully develop. Once these local failures occur, the structure sheds load through discrete fracture events rather than maintaining a smooth plateau response. This interrupts the continuous dissipation of strain energy and contributes directly to the lower SEA measured for the hollow lattice. The figure shows three features that are mechanically important. First, deformation and damage are concentrated in the lower row, which confirms the non-uniform load sharing already visible in the compression sequence. Second, cracks initiate at junctions and crossover regions, which are the locations where bending, local shear, and geometric discontinuity are concentrated in this topology. Third, the exposed strut sections show open internal channels, which confirms that the tested lattice is genuinely hollow and not partially resin filled. These regions control the transition from recoverable mechanism-driven deformation to damage-driven response. Once cracking begins at these sites, the coordinated rotation required for a strong global auxetic response is interrupted and the structure starts to shed load in a non-uniform way. This mechanism is consistent with the stress-drop sequence seen later in the hollow-lattice stress–strain curve in Figure 9. It also matches the wider understanding that node regions in architected lattices are often the most defect-sensitive and have mechanically critical features. That point matters here because the architecture is hollow. The printability envelope and drainage strategy define what can be fabricated, while the post-fracture image in Figure 10 confirms visible hollow channels and therefore supports the interpretation that the measured response belongs to a genuinely hollow lattice rather than a partially resin-filled structure. Defect-aware modelling studies have shown that even small geometric deviations can shift stiffness and failure response in lattices, especially near nodes and thin-wall transitions [42]. The present fracture sequence is therefore mechanically credible for a hollow SLA lattice and supports the observed gap between idealised optimisation and realised lattice performance.

The hollow versus solid comparison in Figure 9 and Table 4 places the auxetic findings in a more application-relevant mechanical context. This indicates that the lower specific energy absorption of the hollow lattice is governed mainly by collapse mode and earlier local failure, rather than by density alone. The hollow and solid lattices have very similar initial stiffness, with only a 3.0 percent reduction in absolute Young’s modulus for the hollow design, despite a 42.4 percent reduction in apparent density. This is a notable result because it shows that hollowing did not simply reduce mass. It redistributed material in a way that preserved the low-strain load path. Once normalised by density, the effect becomes much clearer. Specific stiffness increases by 68.9 percent and specific peak strength increases by 5.2 percent for the hollow lattice, while specific energy absorption decreases by 25.6 percent. The stress–strain curves in Figure 9 explain this combination. The hollow lattice enters a fracture-dominated plateau with distinct stress drops, whereas the solid lattice shows a more stable progression toward densification. This is characteristic of tube-based and hollow-strut lattices, where mass efficiency improves but collapse can become more sensitive to local instability and node failure [43]. The reduction in specific energy absorption is also consistent with hollow-walled lattice studies showing that collapse mode and local wall geometry govern energy absorption more strongly than relative density alone [44]. The present results therefore indicate that the current hollow architecture is already well suited to weight-critical, stiffness-limited applications such as lightweight cores, inserts, and other low-volume polymer components where geometric control and mass-efficient stiffness are important. By contrast, energy-absorbing applications will require local redesign to delay crack initiation and stabilise progressive collapse [45].

The numerical and experimental results also provide a clear basis for the next stage of design development. Table 2 shows that the geometry optimisation is effective at identifying a strongly auxetic region and selecting a high-potential geometry. Figure 8a,b show that the intended kinematics survive fabrication and are still visible at lattice scale. Figure 10 shows that fracture initiates at localised, mechanically critical regions and interrupts full mechanism development. Figure 9 and Table 3 show that hollowing delivers a strong stiffness-to-weight advantage but introduces a fracture-dominated collapse path. The main design challenge is therefore no longer whether the geometry can generate auxeticity. It is how to preserve more of that auxetic response in a finite lattice while maintaining the mass-efficiency benefits of hollow struts. The most direct route is to move from unit cell optimisation alone to lattice-aware optimisation that includes boundary conditions, deformation uniformity, and node-level failure resistance as explicit objectives. This direction is consistent with the broader evolution of auxetic and metamaterial design, where mechanism design, boundary effects, and structural robustness need to be treated together in order to achieve reliable performance in practical components.

## 5. Conclusions

A hollow hybrid re-entrant chiral auxetic lattice was successfully taken from parametric design and numerical optimisation to stereolithography fabrication and experimental validation. The optimised geometry, θ = 15°, L = 3.5 mm, and d = 1.68 mm, retained the intended rotation-driven auxetic deformation mode in both the printed unit cell and the 3 × 3 × 3 lattice. The results show that the auxetic mechanism transfers across scale, but its magnitude is reduced in the finite lattice. At the selected comparison point, the unit cell reached an NPR of −0.68 and the lattice reached −0.29, compared with an optimised numerical unit cell prediction of about −1.17. The observed reduction is consistent with the measured deformation localisation and junction-driven fracture, indicating that realised lattice performance is governed by finite-size effects, load redistribution, and local failure rather than unit cell kinematics alone. The hollow architecture also delivered a clear lightweight performance benefit. Compared with the solid lattice, the hollow lattice showed only a 3.0% reduction in absolute stiffness while reducing density by 42.4%, which increased specific stiffness by 68.9% and specific peak strength by 5.2%. This gain came with a 25.6% reduction in specific energy absorption due to earlier fracture and a more fracture-dominated collapse response. These findings show that the present hollow auxetic design is already well suited to weight-critical, stiffness-limited applications. Although the hollow design did not improve specific energy absorption in its current form, the fracture evidence identifies clear redesign targets at junction and crossover regions for future energy-absorbing variants. The next step is to move from unit cell optimisation to lattice-scale optimisation with explicit treatment of boundary effects, joint behaviour, and wall-thickness distribution so that more of the optimised auxetic response is retained in larger printed lattices.

## Figures and Tables

**Table 1:** Factors and levels used in the design of experiments.

Factor (Parameter)	Symbol	Lower Bound	Upper Bound	Units
Chiral angle	θ	0	30	deg
Re-entrant strut length	L	1.0	4.5	mm
Hollow internal diameter	d	1.5	2.0	mm

**Table 2:** DOE matrix and finite element Poisson’s ratio response.

Experiment No.	θ (Deg)	L (mm)	d (mm)	FEA Poisson’s Ratio
1	0	1.00	1.50	−0.45276
2	30	1.00	1.50	−0.65817
3	0	4.50	1.50	−1.17486
4	30	4.50	1.50	−0.71496
5	0	1.00	2.00	−0.38865
6	30	1.00	2.00	−0.66429
7	0	4.50	2.00	−1.05174
8	30	4.50	2.00	−0.60025
9	0	2.75	1.75	−0.89603
10	30	2.75	1.75	−1.01713
11	15	1.00	1.75	−0.63963
12	15	4.50	1.75	−1.19745
13	15	2.75	1.50	−1.03545
14	15	2.75	2.00	−0.92002
15	15	2.75	1.75	−1.05750
16	15	2.75	1.75	−1.05750
17	15	2.75	1.75	−1.05750
18	15	2.75	1.75	−1.05750
19	15	2.75	1.75	−1.05750
20	15	2.75	1.75	−1.05750

**Table 3:** Key ANOVA findings for the quadratic response surface model of Poisson’s ratio.

C	*p*-Value	Significant (*p* < 0.05)	Meaning	Implication
θ	0.156	No	Weak main effect	Not a dominant factor alone
L	0.000	Yes	Strong main effect	Main parameter controlling response
d	0.068	No	Limited main effect	Secondary tuning parameter
θ^2^	0.017	Yes	Nonlinear angle effect	Needs optimisation
L^2^	0.003	Yes	Nonlinear length effect	Needs optimisation
d^2^	0.043	Yes	Nonlinear diameter effect	Needs optimisation
θ × L	0.000	Yes	Strong interaction	θ depends on L
θ × d	0.672	No	Weak interaction	Minor effect
L × d	0.342	No	Weak interaction	Minor effect

**Table 4:** Mechanical performance comparison of the optimised hollow and solid 3 × 3 × 3 lattices.

Metric	Hollow Lattice (HS)	Solid Lattice (SS)	Units
Young’s modulus	3.22	3.32	MPa
Peak compressive strength	0.306	0.507	MPa
Apparent density	125	217	kg m^−3^
Specific Young’s modulus	25,777	15,258	m^2^ s^−2^
Specific peak compressive strength	2450	2330	m^2^ s^−2^
Specific energy absorption (SEA)	303	407	J kg^−1^

## Data Availability

The original contributions presented in this study are included in the article. Further inquiries can be directed to the corresponding authors.
